# γ-Aminobutyric acid confers cadmium tolerance in maize plants by concerted regulation of polyamine metabolism and antioxidant defense systems

**DOI:** 10.1038/s41598-020-59592-1

**Published:** 2020-02-25

**Authors:** Maryam Seifikalhor, Sasan Aliniaeifard, Françoise Bernard, Mehdi Seif, Mojgan Latifi, Batool Hassani, Fardad Didaran, Massimo Bosacchi, Hassan Rezadoost, Tao Li

**Affiliations:** 1 Department of Plant Biology, College of Science, University of Tehran, Tehran, Iran; 2 Photosynthesis laboratory, Department of Horticulture, College of Aburaihan, University of Tehran, Tehran, Iran; 3 Faculty of Life Sciences and Biotechnology, Department of Plant Sciences, Shahid Beheshti University G.C., Tehran, Iran; 4 KWS Gateway Research Center, LLC, BRDG Park at the Danforth Plant Science Center, Saint Louis, USA; 5 Department of Phytochemistry, Medicinal Plants and Drugs Research Institute, Shahid Beheshti University, G.C., Evin, Tehran, Iran; 6 Institute of Environment and Sustainable Development in Agriculture, Chinese Academy of Agricultural Science, Beijing, China

**Keywords:** Plant physiology, Plant stress responses

## Abstract

Gamma-Aminobutyric acid (GABA) accumulates in plants following exposure to heavy metals. To investigate the role of GABA in cadmium (Cd) tolerance and elucidate the underlying mechanisms, GABA (0, 25 and 50 µM) was applied to Cd-treated maize plants. Vegetative growth parameters were improved in both Cd-treated and control plants due to GABA application. Cd uptake and translocation were considerably inhibited by GABA. Antioxidant enzyme activity was enhanced in plants subjected to Cd. Concurrently GABA caused further increases in catalase and superoxide dismutase activities, which led to a significant reduction in hydrogen peroxide, superoxide anion and malondealdehyde contents under stress conditions. Polyamine biosynthesis-responsive genes, namely ornithine decarboxylase and spermidine synthase, were induced by GABA in plants grown under Cd shock. GABA suppressed polyamine oxidase, a gene related to polyamine catabolism, when plants were exposed to Cd. Consequently, different forms of polyamines were elevated in Cd-exposed plants following GABA application. The maximum quantum efficiency of photosystem II (F_v_/F_m_) was decreased by Cd-exposed plants, but was completely restored by GABA to the same value in the control. These results suggest a multifaceted contribution of GABA, through regulation of Cd uptake, production of reactive oxygen species and polyamine metabolism, in response to Cd stress.

## Introduction

Heavy metal (HM) toxicity is considered a major threat to living organisms. HM-polluted soils derived from increasing geologic and anthropogenic activities have significantly impacted the production of high-quality agricultural crops in certain regions of the world. Plants growing on these soils exhibit reduced growth, photosynthetic performance, and yield^1^. As a naturally occurring HM pollutant, Cd exposure has been documented in most organisms, particularly plants and humans^2^.

World fertilizer consumption is increasing and will eventually reach a point where the drawbacks outweigh the benefits^3^. The same mechanisms that drive improved plant productivity often create side effects such as environmental contamination. Furthermore, some components of fertilizers, especially Cd, accumulate in both body and food chain, where they remain for an extended period and causes adverse health effects^4^. Therefore, fertilizers containing very high levels of Cd (417 mg/kg) threaten human health by accumulation in important crop such as maize^5^. Maize, as one of the most popular cereal grain, is widely cultivated across the world. Maize is also produced at an industrial scale as a key input in various products such as syrups, soft drinks and charcoals^6,7^. Therefore, there is a strong incentive to minimize the toxic effects of Cd in maize.

The mechanism of Cd toxicity in plants is still under investigation though it has been proposed to disturb photosynthetic function via inhibition of oxygen evolution and reduced chlorophyll content^8–10^. Plants exposed to high Cd concentrations exhibit reduced photosynthesis and root cellular metabolism^11–14^. Investigation on PSII function during the photo-activation process confirms that Cd possesses an inhibitory effect through active competition with Ca^2+^ ions in the catalytic center^15^.

**Figure 1:**
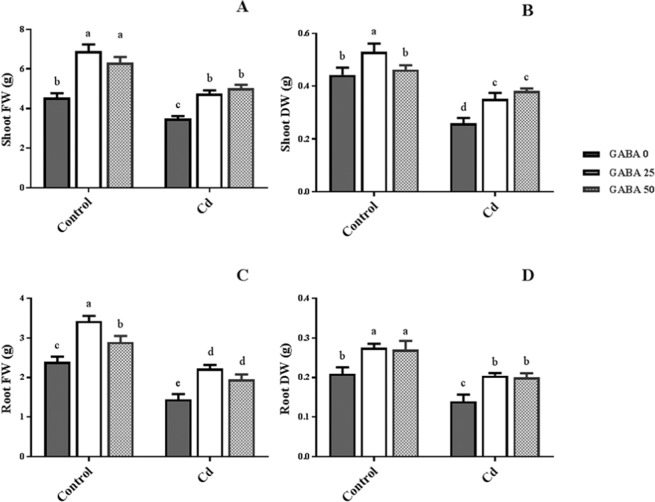
Effects of exogenous GABA (0, 25 and 50 µM) applications on shoot and root fresh (**A**,**C**) and dry weights (**B**,**D**) of maize seedlings exposed to 0 (control) and 250 µM cadmium (Cd).Values are the means of six replicates and bars indicate means ± SEM.

**Figure 2:**
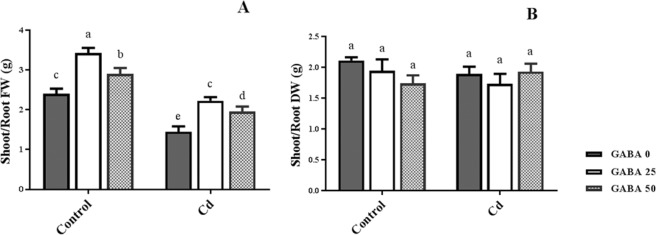
Effects of exogenous GABA (0, 25 and 50 µM) applications on shoot to root fresh [(FW), **A**] and dry weight [(DW), **B**] ratios in maize seedlings exposed to 0 (control) and 250 µM cadmium (Cd). Values are the means of six replicates and bars indicate means ± SEM.

During evolution plants have evolved diverse adaptive strategies to alleviate the negative effects of high Cd concentrations. These adaptations are associated with the production of Cd-sequestering compounds such as phytochelatins or with active Cd export from the cell^16^. Recent studies have revealed a link between low molecular weight thiol (–SH) compound in most plant and Cd toxicity^17^. In different studies, increase in glutathione (GSH) content, as one of the main thiol compound; have been reported after Cd exposure; as it is the precursor for the synthesis of phytochelatins^18^. Detoxification is another general mechanism developed in plants to cope with highly toxic metals and to maintain physiological tolerance. Under stress condition endogenous regulating networks set in motion to maintain a delicate balance between reactive oxygen species (ROS) production and ROS-scavenging pathways. Antioxidants enzymes comprising superoxide dismutase (SOD), ascorbate peroxidase (APX), catalase (CAT) and glutathione peroxidase are commonly present in all intracellular regions and mainly responsible for oxidative burst. Alternatively, chelation of metals by ligands is a possible strategy by which compartmentalization of the ligand-metal into a limited area prevents the free circulation of ions in the cytosol^12^. Notably, in response to HM stresses, plants accumulate signaling molecules that maintain their capacity for growth and development^19,20^. In this regard, plant stress perception is a crucial step for the rapid induction of defense responses^21^, and this perception may be enhanced by stress-derived signals^22,23^. As well-known stress responsive metabolites, polyamines (PAs), a class of biogenic amines with multiple functions that includes putrescine (Put), spermidine (Spd), and spermine (Spm), significantly accumulate in plants exposed to biotic or abiotic stresses^24^. PA involvement in plant-related responses has been well documented as: (i) PA content decreases in stress-susceptible plants^25,26^, though (ii) under stress conditions PA-dependent gene expression is elevated^27^ and (iii) plants treated by exogenous PAs exhibit stress tolerance^28–30^. In plants, L-arginine is decarboxylated by ornithine decarboxylase (ORDC) and arginine decarboxylase (ADC) to generate Put. S-adenosylmethionine decarboxylase generates an aminopropyl group that is subsequently linked to Put by Spd synthase (SPDS) or Spm synthase (SPMS), to generate Spd and Spm, respectively. Conversely, PA catabolism in the cell is carried out by PA oxidase (PAO)^24,31,32^. PAO activity has been revealed in different plant species including maize^33^. Additionally, by-products generated from PA degradation pathways function as high throughput alternative components. A well-known example is GABA^34^. GABA is a four carbon non-protein amino acid found in plants in response to myriad biotic and abiotic stresses^35–41^. GABA accumulation has been reported as a result of exposure to different stress conditions such as high temperature^42^, osmotic pressure^19,43^, cold shock^44^, salinity stress^22^ and HM toxicity^19^. It has been shown that exogenous GABA application can induce stress tolerance in plants. For example, significant increases of heat tolerance in creeping bentgrass (*Agrostis stolonifera*)^39^, cold tolerance in peach fruits (*Amygdalus persica*)^45^, salt tolerance in *Nicotiana sylvestris*^46^ and *Lactuca sativa*^41^ and drought tolerance in perennial ryegrass (*Lolium perenne*)^47^ have been previously reported. The beneficial role of GABA against stress-induced oxidative damage in various plant species has been often reported^41,48,49^. GABA has also been attributed to cytosolic pH regulation since its biosynthesis consumes protons, thereby reducing acidic conditions^50–52^.

Although a number of studies have specifically linked GABA accumulation with stress, there is little information about the specific manner that GABA contributes to HM tolerance in plants. In the current study we have focused on PA metabolism, with the aim to discover the regulatory pathway underlying GABA’s counteractive effect on Cd toxicity in maize plants. Considering the beneficial role of GA'A in biotic and abiotic stress tolerance in plants, this study demonstrates how GABA modulates the plant responses to Cd toxicity with an emphasis on assessing the effects of exogenous GABA on Cd uptake, ROS and PAs metabolism during Cd stress conditions. Our results will pave the way for future studies that manipulate key steps in these metabolic pathways, to induce tolerance against Cd toxicity.

**Figure 3:**
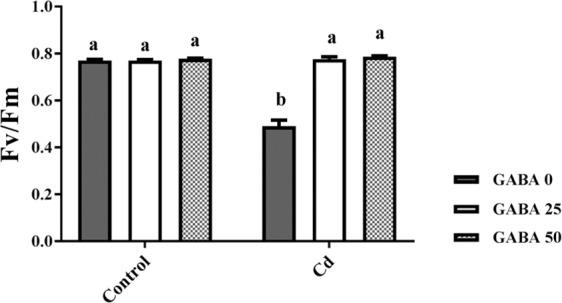
Effects of exogenous GABA (0, 25 and 50 µM) applications on maximum quantum yield of photosystem II (F_v_/F_m_) in maize plants exposed to 0 (control) and 250 µM cadmium (Cd). Values are the means of five replicates and bars indicate means ± SEM.

## Results

### GABA improves growth of maize plants

A significant increase was observed in shoot and root fresh weights in both Cd-exposed and control plants as a result of GABA applications (Fig. 1A,C). Similar effects of GABA were recorded for shoot and root dry weights (Fig. 1B,D). The results omitted from shoot/root ratio of plant’s fresh weight represented a reduction by Cd exposure in compare to the controls, though GABA increased shoot biomass and led to a significant increase in shoot/root ratio in both Cd-exposed an free conditions (Fig. 2A). However, shoot/root dry weight averaged 1.9, resulted in no obvious difference among plants (Fig. 2B). These observations indicate the positive role of GABA on growth improvement under Cd stress conditions. In addition, the stimulatory effect of GABA on plant growth in the absence of Cd treatment is also conclusive.

### GABA improves photosynthetic electron flow in Cd-exposed maize plants

Under control conditions, no significant change was observed in F_v_/F_m_ as a result of GABA concentrations (Fig. 3). Cd treatment caused a considerable (≈50%) reduction in F_v_/F_m_ when compared to the control plants; while in Cd-exposed plants GABA supplementation fully restored F_v_/F_m_ to the similar values as the control plants (Fig. 3). These observations indicate the protective role of GABA against negative effects of Cd on the photosynthetic function of maize plants.

### Inhibitory effect of GABA on Cd uptake and transport

**Figure 4:**
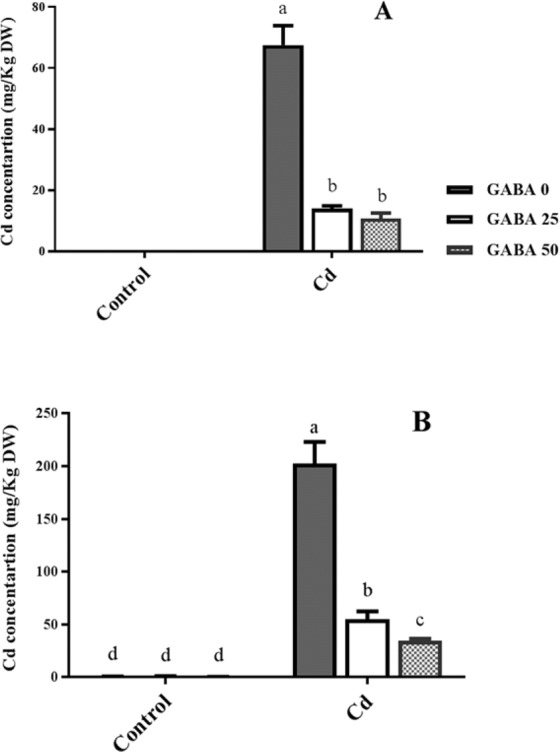
Effects of exogenous GABA (0, 25 and 50 µM) applications on cadmium (Cd) concentrations in shoots (**A**) and roots (**B**) of maize plants exposed to 0 (control) and 250 µM Cd. Values are the means of six replicates and bars indicate means ± SEM.

**Figure 5:**
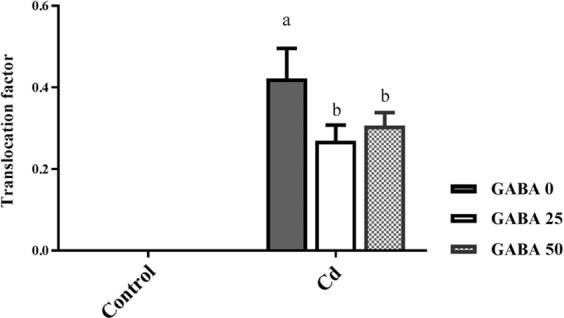
Effect of exogenous GABA (0, 25 and 50 µM) applications on translocation factor of cadmium (Cd) from roots to shoots in maize plants exposed to 0 (control) and 250 µM Cd. Values are the means of six replicates and bars indicate means ± SEM.

Plants grown in Cd-free medium exhibited null Cd amounts in both shoot and root tissues (Fig. 4A,B). Cd concentrations in the shoots of Cd-exposed plants were approximately four times higher than in plants co-treated with Cd and GABA (Fig. 4A). In plants grown under Cd stress, the highest amount of Cd was observed in the roots of plants irrigated with GABA-free solutions. GABA applications at 25 and 50 µM concentrations led to 72% and 82% reduction in Cd content of the root tissues, respectively (Fig. 4B). However, under Cd stress the highest Cd translocation factor (0.42) was detected in plants grown on GABA-free medium while GABA application reduced Cd transfer from root to the shoots (Fig. 5). These observations indicate inhibitory effects of GABA on Cd absorption by the root and on translocation of Cd from root to shoot.

### Antioxidant defense system is powered by GABA against Cd stress

**Figure 6:**
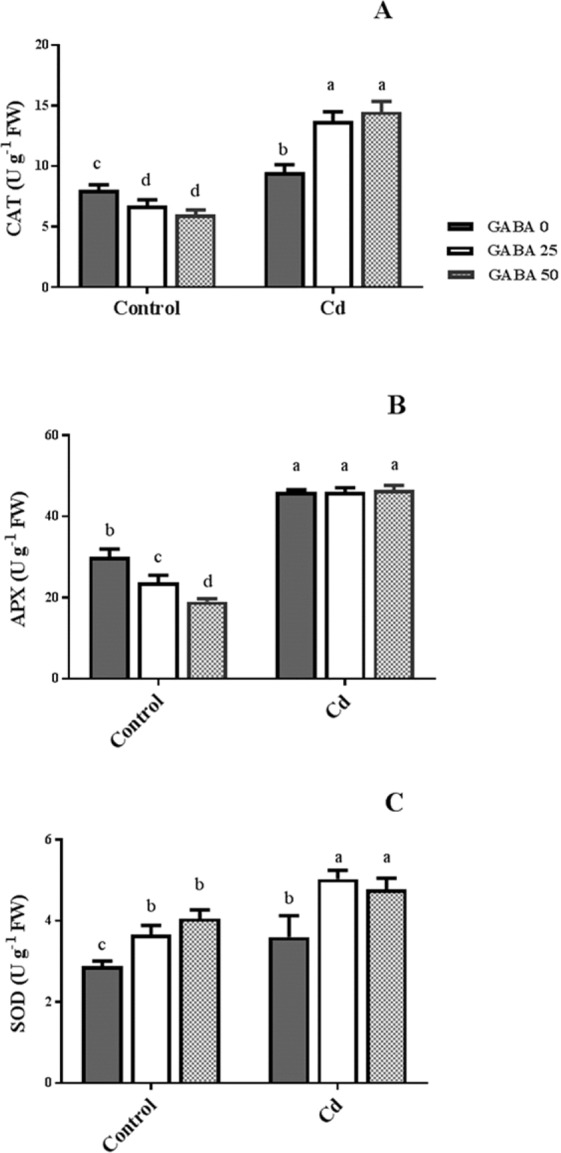
Effects of exogenous GABA (0, 25 and 50 µM) applications on antioxidant enzyme [CAT (**A)**, APX (**B**), and SOD (**C**)] activities in maize plants exposed to 0 (control) and 250 µM cadmium (Cd). Values are the means of six replicates and bars indicate means ± SEM.

GABA reduced CAT enzyme activity in a GABA concentration-dependent manner in control plants, while, Cd-exposed plants exhibited significant increase (30%) in CAT activity when supplemented with GABA (Fig. 6A). Similarly, APX enzyme activity was reduced by GABA application in control plants. Cd in the root medium led to a sharp rise in APX activity irrespective of GABA application (Fig. 6B). GABA exposure elevated (about 30%) the SOD activity in plants grown in both control and Cd conditions. The highest SOD activity was observed in plants co-treated with Cd and GABA (Fig. 6C).

**Figure 7:**
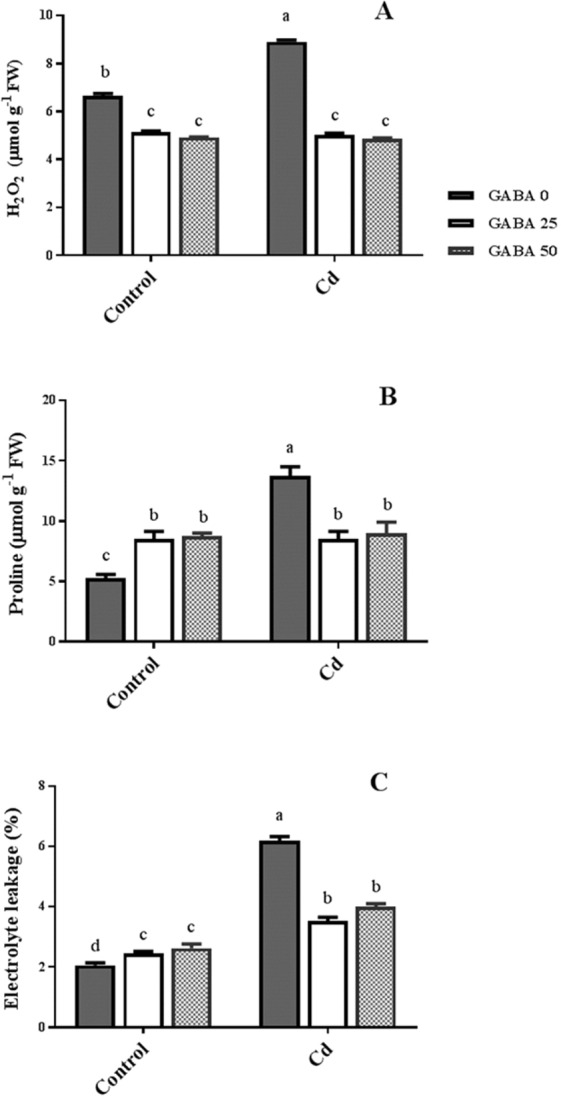
Effects of exogenous GABA (0, 25 and 50 µM) applications on proline concentration (**A**), H_2_O_2_ level (**B**) and electrolyte leakage percentage (**C**) in maize plants exposed to 0 (control) and 250 µM cadmium (Cd). Values are the means of six replicates and bars indicate means ± SEM.

**Figure 8:**
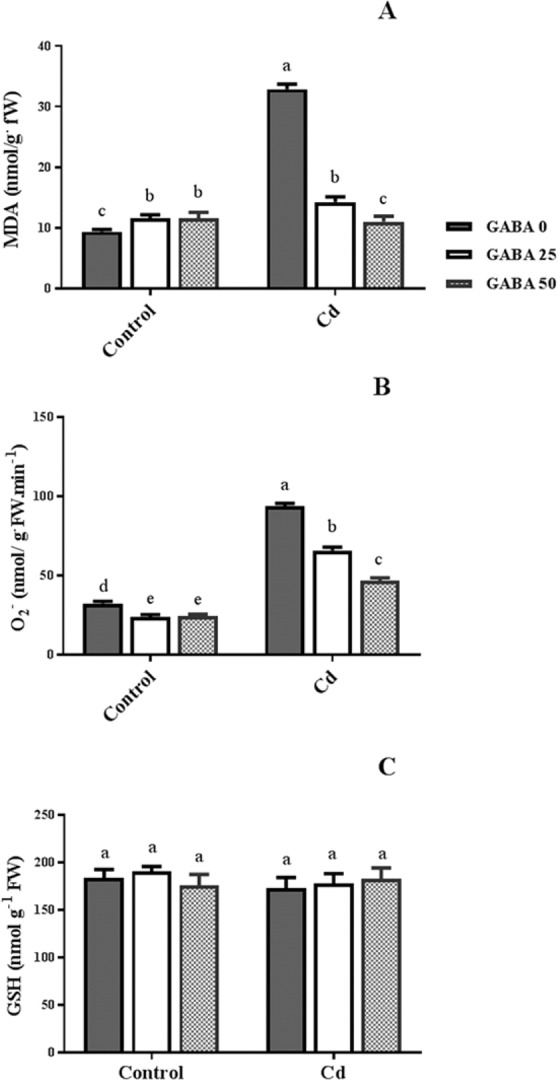
Effects of exogenous GABA (0, 25 and 50 µM) application on malondialdehyde (MDA) (**A**), ${{\rm{O}}}_{2}^{-.}$ (**B**) and glutathione (GSH) (**C**) levels in maize plants exposed to 0 (control) and 250 µM cadmium (Cd). Values are the means of four replicates and bars indicate means ± SEM.

Hydrogen peroxide (H_2_O_2_) content was approximately 30% higher in plants exposed to Cd, versus control plants. However, plants grown under Cd stress maintained the same H_2_O_2_ content as the control set when GABA was applied to the rhizosphere (Fig. 7A). In Cd-free conditions, the proline content of GABA-treated plants was approximately 40% higher than proline level of control plants. In contrast, proline level in plants co-treated with Cd and GABA was approximately 40% lower than its content plants treated with Cd alone. However, the levels of proline in Cd-treated plants following GABA application was similar to that of the control plants (Fig. 7B), implying a change in proline content irrespective of GABA application. In plants grown under Cd stress, the percentage of electrolyte leakage (EL) was three times higher than EL% of control plants. However in stressed plants, application of GABA effectively (up to 50%) mitigated the EL level when compared with EL% in free-GABA exposed plants (Fig. 7C). MDA level was slightly (about 10%) increased by exogenous GABA application in control plants. Cd caused 3.5-fold rises in MDA content. However, it sharply reduced to the same levels in Cd-free plants as a result of GABA application (Fig. 8A). In plants exposed to Cd-free medium, ${{\rm{O}}}_{2}^{-.}$ levels were reduced following GABA application. Although ${{\rm{O}}}_{2}^{-.}$ content was enhanced by Cd, its concentration was reduced (about 30%) in GABA-treated plants in a GABA concentration-dependent manner (Fig. 8B). No change in GSH content was detected following Cd exposure; also GABA showed null effect in both conditions on GSH level in the leaves of maize plants (Fig. 8C).

### Modulation of PAs pathway by exogenous GABA in maize plants

**Figure 9:**
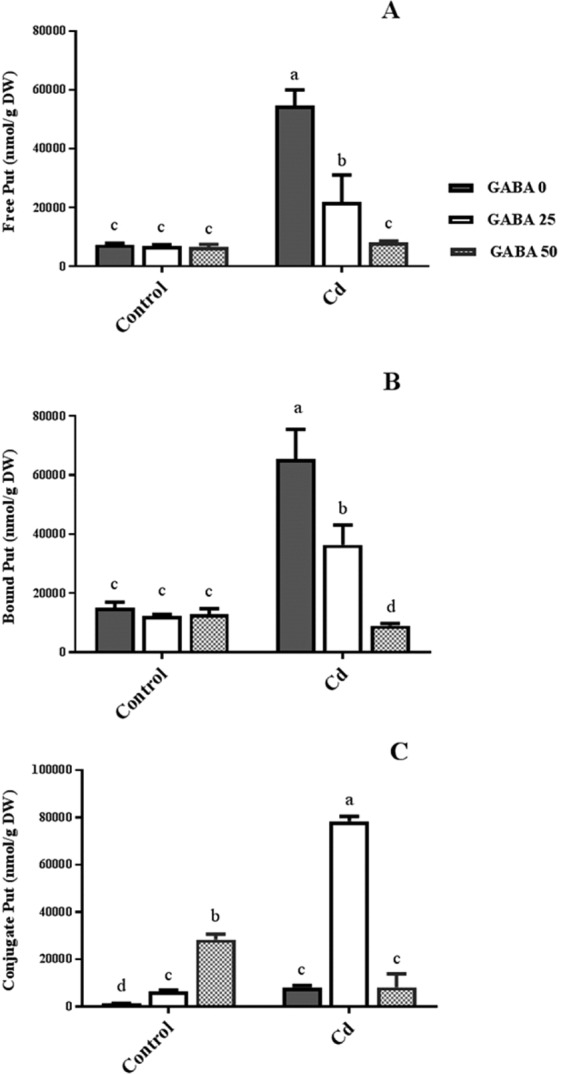
Effects of exogenous GABA (0, 25 and 50 µM) applications on different forms of putrescine (Put) [free (**A**) insoluble bound (**B**) and soluble conjugated (**C**)] contents in maize plants exposed to 0 (control) and 250 µM cadmium (Cd). Values are the means of three biological and three technical replicates and bars indicate means ± SEM.

Free Put (FP) level did not change under non-stress conditions following GABA applications, however under Cd stress, maximum FP content was observed in plants receiving null concentrations of GABA. Under stress conditions, FP contents were decreased in a GABA concentration-dependent manner (Fig. 9A). Treatment with GABA showed no effect on insoluble bound Put (IBP) level in plants grown under normal condition; while Cd application resulted in a five-fold increase in IBP content. IBP level was decreased in a GABA concentration-dependent manner in Cd-exposed plants (Fig. 9B). GABA application increased soluble conjugated Put (SCP) in control plants with a sharp increase (28 fold) observed in the 50 µM GABA treatment. However, the highest increases were detected in stressed plants pretreated with 25 µM GABA (Fig. 9C). According to these results, although GABA showed positive effect on different types of Put levels, the maximum increase was detected in SCP form under Cd stress condition.

**Figure 10:**
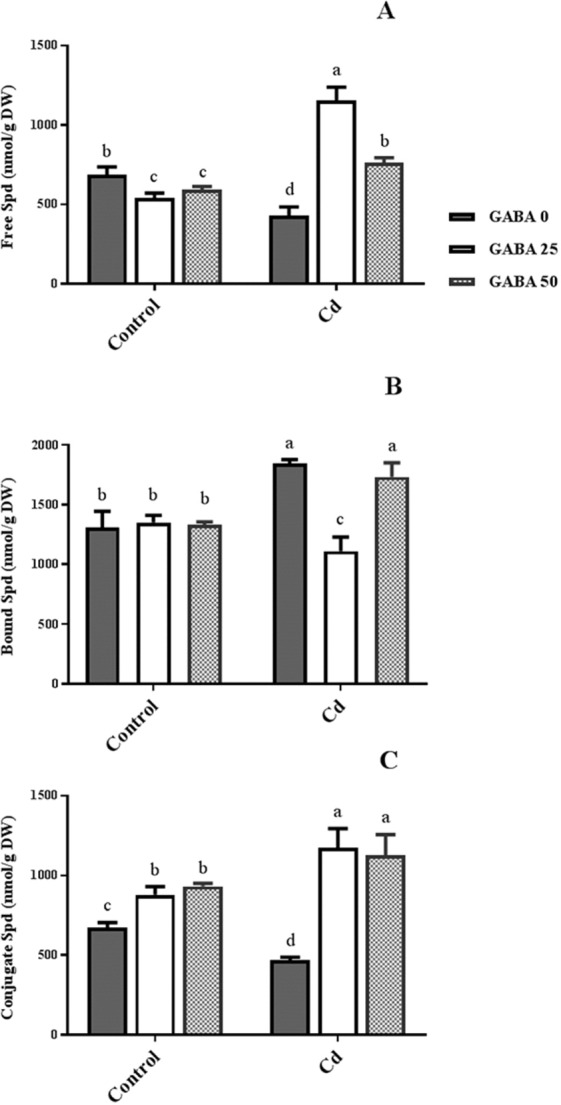
Effects of exogenous GABA (0, 25 and 50 µM) applications on different forms of spermidine (Spd) [free (**A**) insoluble bound (**B**) and soluble conjugated (**C**)] content in maize plants exposed to 0 (control) and 250 µM cadmium (Cd). Values are the means of three biological and three technical replicates and bars indicate means ± SEM.

Free Spd (FS) content was reduced 20% from GABA application under non-stress conditions. Although Cd application resulted in 40% decrease in FS level compared with control plants, 2.7 and 1.7-fold increases were observed in Cd-exposed plants treated with 25 µM and 50 µM GABA, respectively (Fig. 10A). Treatments with GABA had no significant effect on insoluble bound Spd (IBS) in control plants. In contrast, nearly 30% increase in IBS levels were observed in plants treated with Cd only, as well as those co-treated with Cd and 50 µM GABA. (Fig. 10B). Higher concentrations of the soluble conjugated form of Spd (SCS) were detected in both control and Cd treated plants when GABA was applied. However, GABA induced more SCS in Cd-exposed plants when compared to the control condition (Fig. 10C).

**Figure 11:**
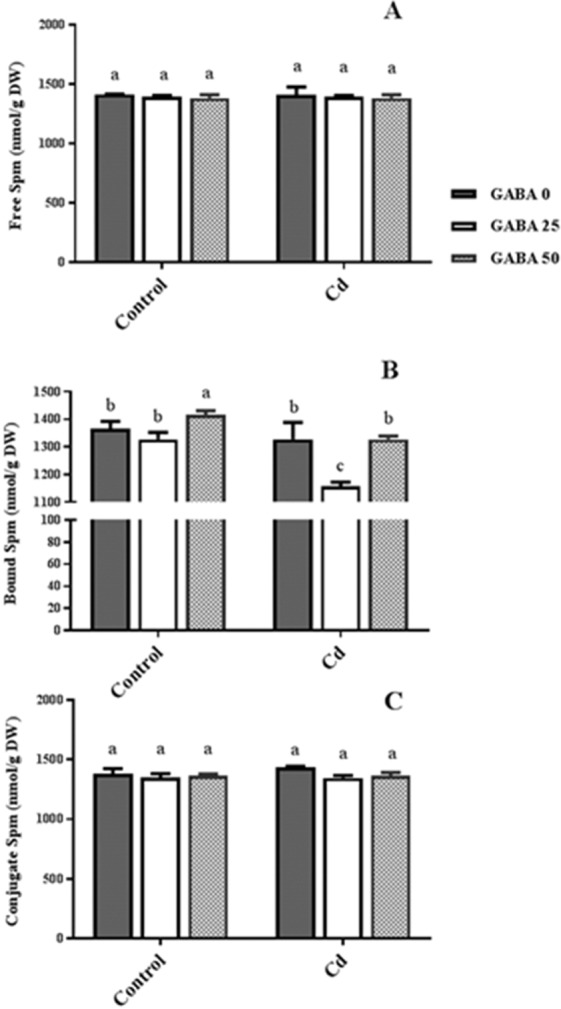
Effects of exogenous GABA (0, 25 and 50 µM) applications on different forms of spermine (Spm) [free (**A**) insoluble bound (**B**) and soluble conjugated (**C**)] contents in maize plants exposed to 0 (control) and 250 µM cadmium (Cd). Values are the means of three biological and three technical replicates and bars indicate means ± SEM.

Insoluble bound Spm (IBSp) in control plants was increased by 50 µM GABA application. However in plant grown under stress condition, 25 µM GABA caused a 23% reduction in IBSp value which resulted in the minimum content of the IBSp form (Fig. 11B). Significant differences in both free and conjugated forms of Spm were not observed in plants as a result of GABA or Cd applications (Fig. 11A,C). Of all the put configurations tested, SCP showed the most increase and change in response to exogenous GABA application against Cd stress.

### Differential regulation of PA-related gene expression by exogenous GABA application

**Figure 12:**
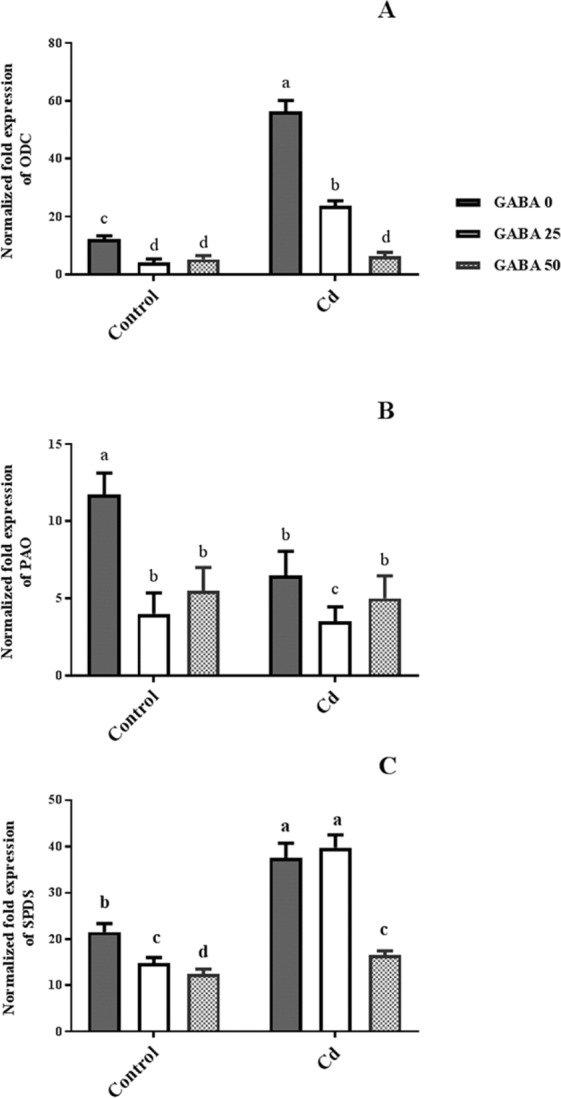
Relative expression levels of ornithine decarboxylase (ORDC) (**A**), PA oxidase (PAO) (**B**) and Spd synthase (SPDS) (**C**) genes in the root of maize plants exposed to different concentrations of GABA (0, 25 and 50 µM) and cadmium (Cd) [0 (control) and 250 µM]. Expression levels are based on the average of three biological replicates that included three technical replicates in maize roots.

Reduced ORDC expression levels (≈70%) were observed in control plants after GABA application. Although similar patterns were also detected in ORDC expression levels under Cd stress, 25 µM GABA treatments caused three-fold enhancement in ORDC gene expression compared with control plants and this increase was four-fold in non-GABA treated plants (Fig. 12A). PAO gene expression was reduced approximately 60% following GABA exposure in control plants (Fig. 12B). Plants subjected to Cd alone showed a 55% decrease in PAO expression levels though this reduction was more observable in plants co-treated by 25 µM GABA. SPDS expression levels doubled as a result of Cd treatment in comparison with control plants; however nearly 30% and 40% reductions were observed in plants treated with 25 and 50 µM GABA, respectively. Under Cd stress, SPDS expression levels increased in plants exposed to 25 µM GABA compared to the control plants (Fig. 12C). These observations imply a positive role of GABA in PA biosynthesis (ORDC and SPDS expression) accompanied by an inhibitory effect on PA catabolism (PAO expression).

## Discussion

GABA is known as a metabolic/signaling substance in plants that accumulates under various biotic and abiotic stresses^35,53,54^. Nevertheless, knowledge about GABA metabolic effect/function against certain stressors such as HMs is still in its infancy. To our knowledge, no study thus far has explored the potential of exogenous GABA applications to alleviate Cd toxicity in plants. The results of this study indicate that exogenous GABA affects the metabolic and physiological responses to Cd toxicity and identifies some metabolic/molecular pathways that underlie this GABA-induced tolerance in maize. In our study Cd-treated plants showed a distinct reduction in vegetative growth when compared to the control plants. Damaging effects of Cd were mitigated by exogenous GABA application (Fig. 1). Negative effects of Cd on plant growth and development have been previously reported in several plant species^12,55,56^. It has been reported that excessive Cd levels cause diverse toxic symptoms such as reduction in root growth^57^ and disturbances in carbohydrate metabolism^58^. These symptoms may principally occur due to an array of interactions at the cellular/molecular levels of the plant cell. Cd can harm plant cells by binding to certain proteins and interfering with their structures or functions^59^. In addition, oxidative damage due to stimulated ROS production could be another damaging effect of excessive Cd levels^60^. In some plant species, however, several mechanisms have evolved to produce HM resistant responses. These all appear to be detoxification strategies which let plants survive and thrive under HM stresses^61^. Although the mechanisms underlying these detoxification strategies are still unclear, this study demonstrates that exogenous GABA reduced ${{{\rm{O}}}_{2}}^{-\cdot }$ levels, as a main ROS, in Cd-exposed plants. ROS fragments DNA and reacts with proteins, and DNA lipid membranes, resulting in damaged cell processes which in extreme circumstances triggers programed cell death^62^. Notably in this study, MDA levels, as the aldehydic lipid breakdown product, were distinctly elevated in Cd-exposed plants following exogenous GABA application, compared to plants supplemented with GABA-free solution (Fig. 8A). In contrast, MDA levels increased following GABA application in control plants thus implying a possible stress-dependent function for GABA. In addition, constant levels of MDA were observed under both stress and control conditions in plants treated with GABA, consistent with the protective role of GABA when plants encounter stressors. This finding in parallel with the increased antioxidant enzymatic activities such as CAT, SOD and APX correlated with GABA application (Fig. 7) reveals a wide role for GABA in controlling oxidative damage under Cd stress. In fact, substantial increases in the activity of SOD in the presence of GABA is indicative of (i) a natural response to elevated levels of ROS due to Cd stress and (ii) a GABA-induced feedback response during Cd stress. Similar to our finding, elevated activity of antioxidant enzymes driven by GABA has been reported in different stress conditions^37,63,64^. An inhibitory role of exogenous GABA on ROS (e.g. H_2_O_2_) production under salt stress in *Caragana intermedia* plants has been shown by Shi and colleagues (2010)^65^. Consistently, in the current study reduced H_2_O_2_ levels as a response to GABA application was recorded under Cd stress condition (Fig. 7A), and associated with enhanced CAT and APX activity. This points us to the down-regulation of H_2_O_2_ production, as relatively long-lived ROS, by GABA as well. Taken together, this data suggesst a role for GABA in building up a feedback response for governing ROS scavenging processes under Cd stress by modulating antioxidant enzymatic activity. Plants are able to govern HM toxicity by phytochelatins, potential ligands including amino acids like proline that have the capacity to form complex with HMs^20^. In current study, proline levels increased in plants exposed to Cd. However after co-application with GABA, proline content was not altered in relative to control plants (Fig. 7B). Proline has been reported to scavenge ROS such as ${{{\rm{O}}}_{2}}^{-\cdot }$^66^. Proline potentially interacts with hydroxyl radicals (${}^{\cdot }{\rm{O}}{\rm{H}}$) and has been offered as a GABA precursor^67^. The influence of NADP^+^/NADPH ratios on proline and ROS metabolism has also been frequently reported^68–70^. In this way, oxidized NADPH mediated by proline is reduced by H_2_O_2_ in the nucleus to serve a steady state redox regulation thereby preventing H_2_O_2_ toxicity. Interestingly, H_2_O_2_, as a component of oxidative burst, has been suggested to induce proline accumulation under stress conditions^71^. This three-membered ring process governed by proline, GABA and H_2_O_2_ can leave two possible scenarios for the constant level of proline observed in our study after co-application of GABA and Cd: reduced biosynthesis of proline due to GABA application as stress relive metabolite, or proline consumption as a GABA biosynthesis precursor. During stress periods, improved photosynthetic function driven by proline has been suggested through regulation of osmotic adjustment^72^. Osmotic adjustment by GABA has also been reported by Renault and colleagues (2011)^43^. In our previous study we showed the positive effect of GABA on photosynthesis performance under salt stress^41^. Consistently, current study indicates an improved photosynthetic functionality by GABA treatment under Cd stress. GABA has been implicated as a modulator of C: N balance in plants, which may indirectly influence photosynthetic performance. When plants are exposed to light, chlorophyll molecules in leaves perceive light as the energy source and converts it to an excited form. To investigate the effects of GABA and Cd on maize plant’ photosynthetic function, chlorophyll fluorescence was measured after the application of different concentrations of GABA and Cd. Photosynthetic performance can be measured via an index called F_v_/F_m_. In our experiment, Cd application significantly reduced F_v_/F_m,_ indicating that the maximum quantum yield of PSII efficiency was dramatically reduced by Cd (Fig. 3A). Interestingly, adding GABA alleviated the negative effect of Cd by extending F_v_ during Cd application. Similar to our finding, in muskmelon (*Cucumis melon*), GABA application improved saline-alkaline resistance by protecting the photosynthetic apparatus and promoting recovery from stress-related photo-inhibition^38^. In addition, protection of the photosynthetic machinery from exogenous GABA application has been shown in creeping bentgrass (*Agrostis stolonifera*) plants suffering from long term exposure to heat stress^39^. Earlier studies reported the increased photosynthetic capacity by exogenous application of PAs in different plant species under various stress conditions^28,73,74^. Previous study conducted in maize and rice plants have revealed that cd-responsive orthologous are conserved in both plants. Among which ZmGAD1 gene, responsible for glutamate decarboxylase (GAD) enzyme (a key enzyme in GABA biosynthesis) has been introduced as a novel Cd-responsive gene and successfully conferred with Cd tolerance in yeast^75^. Here we have focused on GABA modulatory effect on PAs as different line of the tolerance pathway in maize plants. The biological link between GABA and PAs is established by the fact that GABA is interconverted through PAs catabolism^34^. In our study, IBP levels remained constant when both concentrations of GABA were applied to non-stressed plants (Fig. 9B). This can imply that GABA inter-relation with Put formation is mainly due to the stress-induced signals in plant^76^. Higher GABA concentrations (50 µM) reduced final IBP level. These results are indicative of higher GABA concentration having a negative feedback effect on IBP biosynthesis. A similar trend was also obtained for FP in Cd-exposed plants (Fig. 9A). Results obtained from SCP measurement indicated a sharp induction from 25 µM GABA application on Cd-exposed plants (Fig. 9C). This result is consistent with a study from Liu and colleagues (2004) reporting higher FP level in drought-sensitive wheat plants versus induced SCP form in tolerant cultivars^77^. In light of these results, a possible hypothesis could be the conversion of FP to SCP forms modulated by 25 µM exogenous GABA application. Enhanced level of SCP has also been correlated with improved drought tolerance in rice plants^78,79^. However, reduced SCP content has been detected by Yong and colleagues in GABA + PEG-treated white clover plants^80^. This conflict can be explained by dynamic strategies employed by different plant species in response to various stressors. Drought stress mainly refers to curtailed water status in plants while in Cd-stressed conditions plants have the challenge of actively removing excess Cd from the cytoplasm and compartmentalizing it to the vacuoles. In both distinct strategies, elevated Put level has been addressed^80,81^. Moreover, elevated expression of ORDC gene by GABA in this study (Fig. 12A) substantiates the notion of GABA-induced Put accumulation. However, there are limited studies establishing Put as a major cause of such effects. This led us to speculate that plants have potential to address an array of variable responses by using the same metabolic resources. However, there is a gap of sufficient information as how the various forms of similar PAs (Put) are being modulated under different stress-related conditions. GABA-treated plants, particularly at the 25 µM concentration, exhibited an observable enhancement in both FS and SCS contents under Cd stress (Fig. 10A,C), while IBS showed dramatic increase at 50 µM GABA and no significant differences at exposure to 25 µM GABA (Fig. 10B). These data indicate that higher FS and SCS levels are not required to cope with Cd stress. PA biosynthesis and degradation by exogenous GABA has been reported in other plant species under stressful conditions^82,83^. However, in our study increased levels of SCS were observed in both Cd-exposed and control plants. In Cd-exposed plants, SPDS gene expression was elevated (Fig. 12C) and more interestingly, PAO gene expression was inhibited by GABA application (Fig. 12B). This tempts us to speculate that the two effects of GABA–induction of Spd formation and inhibition of self-degradation–are different sides of the same coin. In an opposite sense, reduced Spm content by GABA application could be attributed to the degradation of Spm to produce Spd. Increased level of Spm in GABA-treated plants can be explained by inhibited degradation of Spm by reduced PAO gene expression. These results imply a multifunctional role for GABA under dynamic plant cell responses.

**Figure 13:**
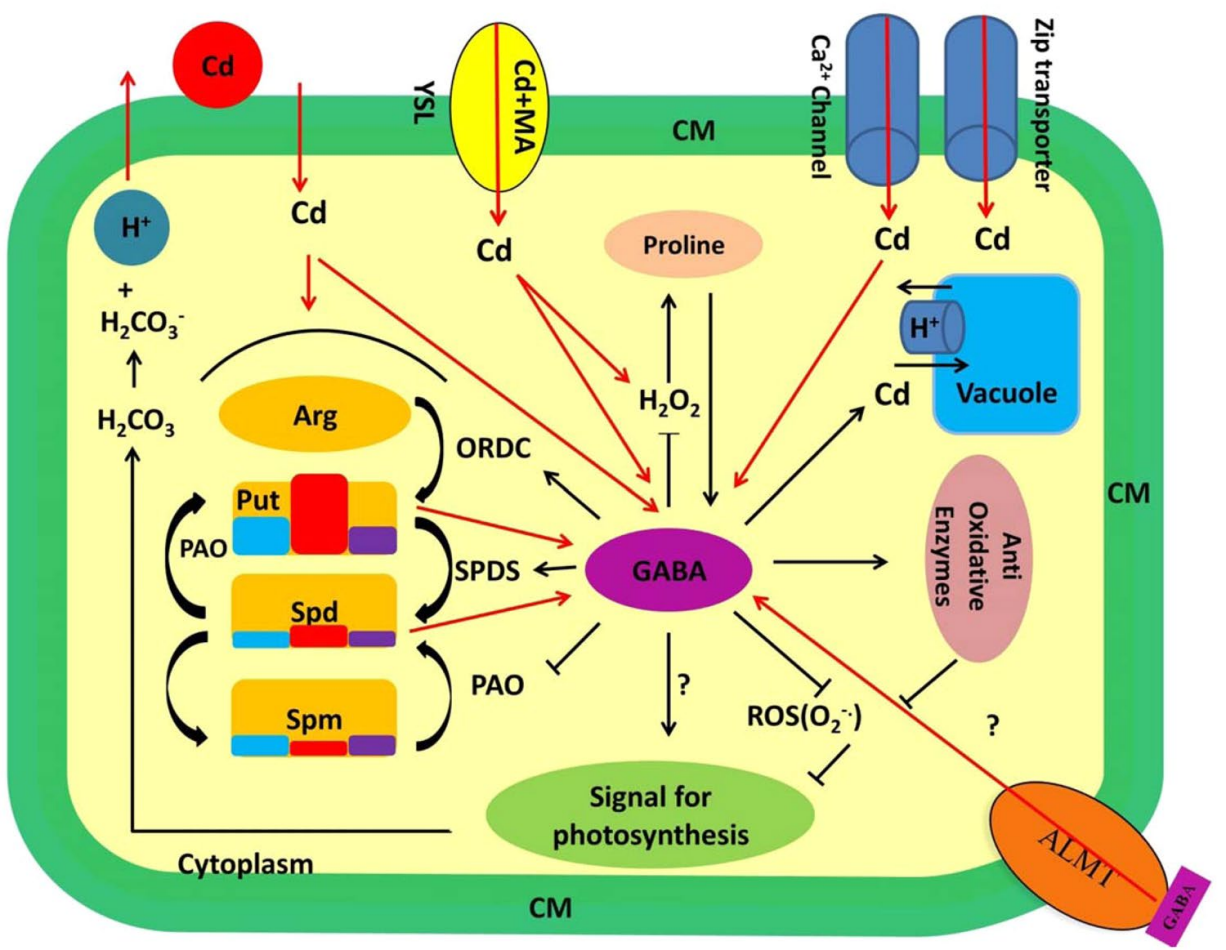
Schematic representation of GABA regulatory role in plant cells under Cd stress. Cd induces PAs and GABA induction in the cell and initiates downstream signaling cues. GABA is involved in different metabolic and molecular processes (black arrows and lines) and in return GABA content in the cell is influenced by diverse pathways (red arrows). PA levels in this scheme have been drawn based on the results obtained from currents experiment in which blue, red and purple boxes representing free, soluble conjugated insoluble bound forms, respectively. Question marks indicate unknown processes. ALTM: Aluminum-activated malate transporter, Arg: Arginine, Cd: Cadmium, CM: Cell membrane, GABA: Gama amino butyric acid, MA: Mugineic acids, ORDC: Ornithine decarboxylase, PAO: Polyamine oxidase, Put: Putrescine, Spd: Spermidine, Spm: Spermine, SPDS: Spermidine synthase, YSL: Yellow Stripe 1-Lik protein.

Several lines of evidence concluded that Cd exposure results in accumulation of thiol compounds in living organisms. Sulphydryl groups of thiols (e.g. GSH) potentially bind to the HMs like Cd and lead to efficient metal sequestration^84^. However our finding is in disagreement with the assumption that Cd tolerance involves an increase of GSH. Our data showed that in maize plants GSH content does not change by Cd exposure and GABA also manifested null effect on GSH level. Previous findings have also shown that the activity of γ-glutamyl-cysteine synthase, a rate limiting enzyme in GSH metabolism, in tolerant plants is lower than its activity in susceptible ones^18^. In a same fashion, transgenic *Arabidopsis* plants harboring bacterial γ-glutamyl-cysteine synthetase resulted in Cd sensitivity^85^. These findings combined with our result let us to speculate that Cd tolerance in different plants may rely on various mechanisms. GSH can “transiently” increase by abiotic stresses such as Cd shock^86^. Alternatively, plants employ largely variable strategy to commit excessive Cd level. One example can be the mechanism that inhibits Cd absorption or translocation in plants. At the cellular level, Cd enters plants mostly through root epidermal cells. This entrance is associated with three well-known steps including, (i) Cd exchange with H_2_CO_3_^−^-released H^+^, (ii) recruitment of the Fe^2+^, Zn^2+^ (belong to ZIP family transporters) such as Yellow Stripe 1-Lik (YSL) protein and Ca^2+^ channels and iii) formation of metal ligand complexes with mugineic acids (MA) in soil and being authorized for entrance^1^ (Fig. 13). Upon entry, long distance translocation of Cd is governed by the xylem loading process mediated by HM P1B-type ATPases enzymes^87,88^. In our current study, Cd uptake by maize plants was significantly reduced by application of GABA, particularly at the 50 µM concentration (Fig. 4). GABA alleviated the Cd level in two district steps comprising uptake from soil and transfer to the shoot. Although, it is hard to determine specific function for GABA in Cd uptake and translocation processes. In this regard, one possible mechanism could be GABA-mediated Cd chelation, which would distance the Cd from the root system. Alternatively, GABA may change the osmotic pressure by regulation of cytosolic pH^50,89,90^, leading to lower Cd absorption at cellular level, and ultimately reduced Cd concentration in the cell. GABA may regulate enzymatic activities to inhibit intra-plant Cd transfer, thereby limited concentration in shoot. This is consistent with our finding as evident from shoot to root ratio that implies higher inhibitory effect of Cd in root growth. To date, knowledge about GABA interaction with certain receptors has been limited to the plasma membrane localized aluminum-activated malate transporter, ALMT, which functions as a GABA receptor^51^. Although this study suggested an inhibitory effect of GABA on Cd uptake by maize plant, a more defined mechanism/s of Cd uptake regulated by GABA still remains to be discovered.

Our results indicate that from early stage, Cd in the rhizosphere can negatively influence growth and development. However, positive effects of GABA on growth parameters in both control and Cd-treated plants are conclusive. Current results manifested a generic role for GABA in improvement of the Cd-borne defects in plants ranging from early growth to cell metabolism ultimately resulting in the protection of photosynthetic functionality. Multifaceted role of GABA was defined by concerted regulation upon ROS production and Cd uptake. Along with Cd stress, GABA conferred positive effects on PA biosynthesis, conversion and gene expression, though in this respect the intermediary role of GABA or its direct effect are still under debate. In light of GABA exposure, stress-related metabolite, ion hemostasis and maintenance of plant cell membrane integrity addressed dynamic responses induced by GABA and imply an impressive function for that (Fig. 1). However, our results illustrate a wide range of roles, which strongly implies a central role for GABA, in plant programmed responses to Cd toxicity. A broader view will be provided by analyzing the role of GABA associated with Cd perception, reverse genetic studies on GABA related pathways, fundamental studies on GABA/phyohormone crosstalk and a closer examination on the effect of GABA on cell membrane ion channels.

## Methods

### Plant materials and growth conditions

Maize (*Zea mays*) seeds were sterilized with 0.5% sodium hypochlorite for 5 minutes and soaked for 6 hours in distilled water at room temperature. Seeds were sown in pots (20 × 30 cm) filled with equal volumes of perlite and cocopite and grown under controlled conditions (light/dark regime of 16/8 hour at 25/20 °C of day/night cycles and relative humidity of 50%). Uniformly germinated seeds were selected for chemical treatment in the greenhouse. Seven day-old seedlings were irrigated with half strength Hoagland solution containing 0, 250 µM Cd; and 0, 25 and 50 µM GABA for three weeks. Irrigation was performed two times per week; once with solution contained Cd and GABA the rest with the half strength Hoagland solution. Every other week, root medium was washed off to preclude any additive effects of Cd accumulation.

### Determination of plant growth parameters

To determinate fresh weight, root and shoot samples were washed off with water to remove soil and blotted gently with soft paper towel to remove any free surface moisture. Fresh weights were determined immediately and dry weights were measured after drying in an oven at 60 °C for 72 h.

### Maximum quantum efficiency of photosystem II (F_v_/F_m_)

Youngest fully developed leaves were used for measuring F_v_/F_m_ in a chlorophyll fluorescence imaging system (Handy FluorCam FC 1000-H; Photon System Instruments, Brno, Czech Republic.(Intact, attached leaves to the plants were dark-adapted for 20 minutes and immediately were used to measure F_v_/F_m_. F_v_/F_m_ was calculated using a custom-made protocol^91–93^. Leaf samples were exposed to short flashes in darkness followed by a saturating light pulse (3900 µmol m^−2^ s^−1^) causing reduction of primary quinone acceptor of photosystem II. The fluorescence data obtained during short flashes in darkness (F_0_) and during the saturating light flash (F_m_) were used to achieve the variable fluorescence (F_v_ = F_m_ − F_0_). The F_v_/F_m_ value was calculated using version 7 of FluorCam software.

### Determination of antioxidant enzyme activity

To extract and measure the activity of antioxidant enzymes, the leaf tissues were powdered by liquid nitrogen and enzyme extraction was performed according to Sariam *et al*.^94^. To measure CAT enzyme activity, 0.5 g of leaf powder was mixed in 10 ml of 0.1 M phosphate buffer (pH:7.5) containing 0.5 ml of EDTA. To extract APX, 0.5 g of powdered leaf was mixed in 10 ml of 0.1 M phosphate buffer (pH:7) containing 0.5 mg of ascorbic acid. The mixture was filtered using soft cloth and the solution was strained and transferred to the centrifuge tubes. The solutions were centrifuged for 15 minutes at 4 °C with 20,000 g. Transparent extractions (supernatant) were used to evaluate the enzyme activity. The activity of CAT enzyme was measured according to the modified method described by Díaz-Vivancos *et al*.^95^. The reaction was started by adding 100 μl of enzyme extraction and decrease in the absorbance of H_2_O_2_ during 1 minute at 240 nm. One CAT unit was considered as the amount of enzyme needed to oxidize 1 mM H_2_O_2_ per minute.

APX enzyme activity was measured according to Nakano *et al*.^96^. APX reaction consisted of 50 mg phosphate buffer (pH:7), 0.5 mM ascorbic acid, 0.1 mM H_2_O_2_ and 100 μl enzyme extraction. APX activity was calculated based on the reduction of ascorbic acid absorption per minute at 290 nm wavelength. One unit of activity of APX was considered as the amount of enzyme necessary for the oxidation of 1 ml of ascorbic acid per minute. The activity of the enzymes was expressed as a specific activity by mg of enzyme unit/fresh leaf weight.

The activity of the SOD enzyme was measured by Giannopolitis *et al*.^97^. The reaction solution contained 13 mM methionine, 75 mM Nitroblue-tetrazolium (NBT), 2 mM riboflavin, 50 mM phosphate buffer (7.8 ppm). The solution was placed under a 15-watt fluorescent lamp with a light intensity of 1000 lux and reaction started by the turning on the fluorescent lamp and left for 10 minutes. The reaction was terminated by turning off the lamps. The reaction solution was coated with black cloth to measure absorbance. The absorbance was measured at 560 nm. One of the samples was not exposed to light and considered as a control. SOD activity was considered according to the amount of enzyme required to 50% inhibition of the photochemical recovery of nitroblute tetrazolium chloride and was calculated based on Asado *et al*.^98^.

### Measurement of EL percentage

EL percentage was measured by using 0.5 cm leaf discs. Leaf discs placed in tubes containing 10 ml of deionized water and incubated for 24 hours at 25 °C on a rotary shaker; first electrical conductivity of the solution was recorded as C1. Second electrical conductivity was determined in 20 minutes autoclaved samples after equilibration at 25 °C and labeled as C2. The EL was calculated based on the ratio between C1and C2^99^.

### Determination of H_2_O_2_ level

Leaf samples homogenized in the reaction mixture containing 0.5 ml of TCA (0.1%), 0.5 ml of K-phosphate buffer (100 mM) and 2 ml reagent (1 M KI w/v in fresh double-distilled water). The standard reaction was prepared by the same solution in the absence of leaf sample. The reaction was developed for 1 hour in darkness and the absorbance was measured at 390 nm. H_2_O_2_ level was determined according to the standard curve prepared with known concentrations of H_2_O_2_ levels base on the method described by Patterson *et al*.^100^.

### Measurement of superoxide anion and lipid peroxidation

Superoxide anion (${{{\rm{O}}}_{2}}^{-\cdot }$) content was measured following the method of Elstner *et al*.^101^. To measure the lipid peroxidation, a testosterone (TBA) test, which identifies MDA as the final product of lipid peroxidation was conducted according to Heath *et al*.^102^. To do so, 0.1 g of fresh leaf tissue was homogenized in 2 ml TCA 0.1% (w/v). The resulting solution was centrifuged at 12000 g for 15 min, 0.5 ml of supernatant were added to 1 ml of 20% TCA solution containing 0.5% TBA. The solution was placed in boiling water for 30 minutes and the reaction was stopped by placing the samples in the ice. The absorbance of the resulting solution was measured at 532 nm and the absorption of other non-specific pigments was also read at a wavelength of 600 nm. Determination of MDA concentration from a shutter ratio of 155 nM^−1^cm^−1^ in terms of nanomol per gram of fresh tissue was calculated using the following equation.

$${\rm{MDA}}=\frac{(\text{A532}-\text{A600})}{155}\times 1000$$

### Measurement of proline content

Proline content was measured according to Bates *et al*.^103^. In this method, a ninhydrin solution was prepared and 1.25 g of ninhydrin were weighed and combined with 30 ml of glacialacetic acid and 20 ml of phosphoric acid 6 M. After dissolution, it incubated at 4 °C for 24 hours. To measure proline, 0.1 g fresh weight sample was weighed and centrifuged in 2 ml of sulfosalicylic acid 3%. Samples were centrifuged for 10 min with 8000 g. 400 microliters of ninhydrin was added to equal volume of the extract and ultimately combined with 400 μl of glacial acetic acid (100%). The solutions were incubated at 100 °C for 1 hour and were transferred to the ice. After cooling down, 800 μl of toluene was added to the sample and vortex vigorously to separate the distinct phases. Finally supernatant (red solution) was removed and read on a spectrophotometer at a wavelength of 520 nm. The amount of proline was calculated according to the following equation:

$$\mu \text{mole}\,{\rm{proline}}/{\rm{g}}=[(\mu {\rm{g}}\,{\rm{proline}}/{\rm{ml}}\times {\rm{ml}}\,{\rm{toluene}})/115.5\,\mu {\rm{g}}/\mu \text{mol}]/[({\rm{g}}\,\text{sample}/5)]$$

### Measurement of GHS content

Fresh leaf samples (0.5 g) were homogenized in 2 ml of 5% sulphosalicylic acid at 4 °C. The homogenate was centrifuged at 10,000 × g for 10 min. 600 µl of 100 mM Phosphate buffer (pH 7.0) and 40 µl of 5′5′ dithiobis-2-nitrobenzoic acid (DTNB) were added to 500 µl of supernatant. The absorbance was read after 2 minutes at 412 nm^17^.

### HPLC analysis of Pas

Extraction of PAs was carried out with a few modifications according to Hassannejad *et al*.^104^. All steps were carried for three replicates and three samples per replicate. 0.2 g of the fresh root tissue was homogenized with 1 μl of perchloric acid (PCA) at 5% volumetric concentration and centrifuged for 35 min at 4 °C with 15000 g. Precipitates were used for insoluble bound PAs extraction and supernatants were separated for extraction of soluble conjugated and free PAs. To retrieve all soluble conjugated PAs, the precipitates were washed three times with PCA. Final sediment were supplemented by 1000 μl of PCA and centrifuged for 35 minutes with 15000 g. Since bound PAs are insoluble in PCA, 1 ml of NaOH (1N) was added to the residual sediment and centrifuged with 21000 g for 30 minutes. Subsequently, 100 μl of the last solution (for analyzing the insoluble bound PAs) and 100 μl of the primary supernatant (for the analysis of the conjugated PAs) was placed into the separate test tubes and 200 μl of HCl (6 Normal) were added to start hydrolysis process. Tubes were closed by a flame and placed in an oven at 110 °C for 18 hours. In order to evaporate the acid, the flamed lids were broken and again placed in an oven for 18 hours at 80 °C. Further, the same procedure was applied to extract all types of PAs. To do so, 100 μl of each solutions containing free, soluble conjugated and the insoluble bound PAs was placed in a separate test tubes and covered with foil. 200 μl Na_2_CO saturated solution was added to each tube. 400 μl of dansyl chloride (concentration of 5 mg/ml) was added to all tubes and incubated for 90 minutes in 60 °C. Subsequently dansyl chloride was removed from the solution and 200 μl of proline (concentration of 0.1 g/ml) was added to each tube and left at 60 °C for 45 minutes. Later, 500 μl of toluene was added to each tube and vortex for 30 seconds. After few minutes different phases performed and the PAs phase was slowly removed and placed in −20 °C. The concentration of all PAs was measured by HPTLC (CAMAG, Switzerland) and winCATS software 1.2.2. The blotting was done on the Merck (60 F254 TLC) papers covered with silica gel. Length of 4 mm and a gap of 10 mm were considered for each band. Ultimately measurement was performed using phase containing cyclohexane and ethyl acetate (V/V 5:4; in which 4 units of cyclohexane with 5 units of ethyl acetate was mixed). Chromatogram scanning was done at two wavelengths of 245 nm and 366 nm.

### RNA extraction and quantitative real-time polymerase chain reaction (qRT-PCR)

Total RNA was isolated from maize roots and treated with DNAse I using Qiagen plant RNAeasy kit (Qiagen; according to the manufacture’s instruction). RNA concentration was quantified by NanoDrop spectrophotometer. cDNA was synthesized from 1 μg of total RNA using an i-script cDNA synthesis kit (Bio-Rad, Hercules, USA) as described in the manufacturer’s protocol. Quantitative RT-PCR was performed using SYBR green based detection (Bio-Rad, Hercules, USA). Experimental setup and execution was conducted using a MyIQ optical cycler, according to protocol provided by the manufacturer (Biorad, Hercules, USA). Data analysis was performed using BioRad iQ5 software (BioRad). Baselines were set at 100 RFU to calculate Ct values. Refrence primer sequnece was obtained from membrane protein PB1A10.07c acording to Manoli *et al*.^105^. Primer sequences are listed in Table 1.

**Table 1:** Primers used in this experiment.

Gene	Gene no.	Primer sequence (5′-3′)	Tm (°C)
PAO	NM_001111636	GCAAGTACCATGTCCAGGG	59
		CGAGGGAACATGGCTGTCA	
ORDC	XM_008672579	CATGGACCACAAGGCTCC	58
		GTCGAAGACGAGCCAGTC	
SPDS	NM_001112372	CGAAAGAATCAGTGTCAGAAC	59
		C GTGCGGTGTCAGCAAAAGC	
Reference		TGTACTCGGCAATGCTCTTG	60
		TTTGATGCTCCAGGCTTACC

### Measurement of Cd concentration

Dried root and shoot samples were weighed and overheated by 700–500 °C for 3 hours and digested in 5 ml of nitric acid and left for 18 hours. The solutions were filtered by Whatman filter paper and diluted with distilled water up to volume of 10 ml. The Cd absorption was measured by Schimadzu atomic absorption/flame emission. In every sample Cd concentration was calculated based on the standard curve creating by a range of defined Cd concentrations. The following equation was used to calculate the Cd content (mg/kg dry weight) of each sample^106^.

$$\begin{array}{c}{\rm{Cd}}\,{\rm{content}}\,{\rm{in}}\,{\rm{root}}\,{\rm{or}}\,{\rm{aerial}}\,{\rm{part}}\,({\rm{mg}}/{\rm{kg}})\\ =\,[{\rm{Cd}}\,{\rm{concentration}}\,{\rm{of}}\,{\rm{tissue}}\,({\rm{mg}}/{\rm{L}})\times {\rm{final}}\,{\rm{volume}}\,{\rm{of}}\,{\rm{sample}}\,({\rm{ml}})]/{\rm{Dry}}\,{\rm{weight}}\,{\rm{of}}\,{\rm{tissue}}\,({\rm{g}}).\end{array}$$

Cd translocation factor was calculated according to Rezvani *et al*.^107^.
